# Functional exploration of taro starch (*Colocasia esculenta*) supplemented yogurt

**DOI:** 10.1002/fsn3.3358

**Published:** 2023-04-28

**Authors:** Muhammad Shaheryar, Muhammad Afzaal, Farhana Nosheen, Ali Imran, Fakhar Islam, Rabia Noreen, Umber Shehzadi, Mohd Asif Shah, Adil Rasool

**Affiliations:** ^1^ Department of Homecnomics Government College University Faisalabad Pakistan; ^2^ Department of Food Science Government College University Faisalabad Pakistan; ^3^ Department of Clinical Nutrition NUR International University Lahore Pakistan; ^4^ Adjunct Faculty University Center for Research & Development Chandigarh University Mohali Punjab India; ^5^ Department of Management Bakhtar University Kabul Afghanistan

**Keywords:** functional, nutritional, syneresis, taro starch, yogurt

## Abstract

Stabilizers are essential components of manufactured products such as yogurt. The addition of stabilizers improves the body, texture, appearance, and mouth feel of yogurt while also preventing technical defects such as syneresis. A study was conducted to optimize the concentration of taro starch in yogurt. The yogurt was fortified at different concentrations of taro starch. Taro starch levels were 0%, 0.5%, 1%, 1.5%, 2%, 2.5%, and 3%, with different storage times (0, 14, and 28 days). The Tukey honesty test was used for mean comparison (*p* < .1). The results of the study showed that maximum moisture and protein content was taken by using 0.5% taro starch and stored for 0 days while maximum fat % was attained in 1.5% taro starch treatment and storage time was 0 days. The maximum water‐holding capacity was increased by adding 1.5% taro starch under 14 days' storage time. Water‐holding capacity started decreasing with the increasing taro concentration. The acidity of yogurt started increasing with the increasing taro starch and the maximum acidity was taken at 2.5% taro starch concentration. The viscosity of the yogurt was maximum at 2% taro starch. As far as it concerned, sensory evolution, aroma, and taste started changing with the increasing taro starch concentration and increasing storage time. The study's goals were to optimize the taro concentration for stabilizing the yogurt synthesis and to probe the impact of taro starch on the physiochemical attributes of yogurt.

## Graphical abstract

The results of the study showed that maximum moisture and protein content was taken by using 0.5% taro starch and stored for 0 days while maximum fat percentage was attained in 1.5% taro starch treatment and storage time was 0 days. The maximum water‐holding capacity was increased by adding 1.5% taro starch under 14‐day storage time. Water‐holding capacity started decreasing with the increasing taro concentration.

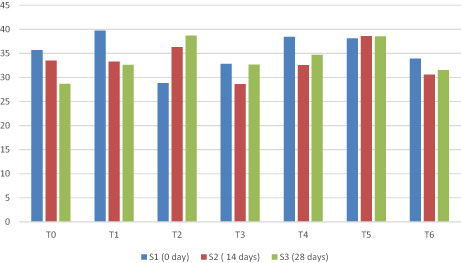

## 1 INTRODUCTION

Milk contains the nutrition that the body needs, including the major nutrients of water, fat, protein, and carbohydrates, as well as the minor nutrients of minerals, enzymes, vitamins, and dissolved gases, it is a necessary sustenance for humans. Milk is readily perishable because the nutrients present in milk act as a hospitable environment for the growth of microorganisms. One strategy to deal with this is to diversify milk processing by fermentation technology to produce yogurt (Gemechu, 2015). Yogurt is a dairy product with a milk casein coagulation pH of about 4.6, and the lactic acid bacteria *L. bulgaricus* and *S. thermophilus* are used in the synthesis of yogurt (Arioui et al., 2017). Many species of BAL that are good probiotics, like *L. acidophilus*, may survive under low pH and adhere to colonize the intestine, improving function and nutritional value. The benefits of yogurt include weight control, probiotics, and immunological impact. They can also aid lactose‐intolerant individual who struggles to digest milk and yogurt's flaw is that throughout production, its ability to hold water decreases, necessitating the use of a binding agent (Pundir et al., 2013). Mckinley (2005) narrated that yogurt is a dairy product that is made from lactic acid fermentation by using *S. thermophilus* and *L. delbrueckii*. Lactic acid bacteria have several known and possible advantages, including the ability to improve lactose digestion, prevent and treat diarrhea, and boost the immune system, which helps the human body fight disease and to validate the role of lactic acid bacteria in antitumor effects, hyper‐cholesterol effects, preventing male genital infections, relieving bowel movement, and treating food allergy, more research must be conducted (Hematyar et al., 2012; Zubair et al., 2023). *C. esculenta*, also known as taro or Arvi, belongs to Araceae family and total production of Arvi is around 9.22 million tonnes around globally. Because they contain minute, easily digestible starch granules that are often in the 70%–80% range, arvi corms are acknowledged to be a high source of starch (Ammar et al., 2009). Arvi is a large source of fat and oil and has a high carbohydrate and calorie content, but it is also poor in fiber. Carotene, riboflavin, thiamine, ascorbic acid, and nicotinic acid are all present in significant amounts. It contains a sizable amount of phosphorus, zinc, and magnesium. Taro (Arvi) cormels and corms are high in commercial starch and flour (Aboubakar et al., 2008). Yogurt is an ancient milk product around the world and is widely used in the world due to its numerous characteristics and high nutritional attributes. The main issue faced by the yogurt industry during production and maintenance is ensuring yogurts’ stabilization and consistency. Yogurt stabilizers are used to upgrade the flavor, texture, and appearance of yogurt, while reducing syneresis (El‐Said et al., 2014).

One of the regional resources that have the potential to be changed into a novel type of binding is the starch found in taro plants (*C. esculenta*). Because they increase the ratio of total solids, develop the viscosity and taste characteristics of the product, and prevent or reduce whey separation during storage, stabilizers are critical in the manufacturing of dairy products (Malik et al., 2012; Sulistyowati et al., 2014). Stabilizers contain a variety of secondary functional characteristics that have been identified, but their impacts on physical, chemical, and sensory properties have not yet been assessed (Imeson, 1999). There are several different sources of stabilizers some are plant origin that is thought to be the least expensive and included the most prevalent, such as maize starch, while others, such as gelatin, are from animal sources. Some of them are synthetic (like carboxymethyl cellulose) (Alakali et al., 2008; Pang et al., 2017). Stabilizers are used as a thickener, gelling agents, syneresis regulators, and emulsifiers in food industry and also control the release of taste and fragrance (Lucey, 2002; Mistry & Hassan, 1992; Nikoofar et al., 2013). To achieve the ideal viscosity and sensory properties, prevent/reduce wheying‐off during distribution and storage, and increase the ratio of total solids, stabilizers must be used in processed dairy products like yogurt. Stabilizers come in a variety of forms, including those made of natural and manmade materials (such as carboxymethyl cellulose) (Islam et al., 2023). Plant‐based stabilizers like maize starch, which are also among the most common, are among the least expensive stabilizers. Starch is used in the dairy industry because of its efficiency as a thickener, its ability to improve texture, and its ability to increase consumer appeal (Zhao et al., 2009). Sweet potato, potato, and chestnut starches are commonly used in yogurt in amounts ranging from 0.25% to 1%. We are not aware of any research evaluating the effectiveness of starches from sources with noticeably different amylose contents, such as tubers, cereals, or legumes, in the manufacturing of yogurt, even though starch is now employed in this process. During and after storage, nonfat yogurt undergoes sensory and rheological alterations (Alakali et al., 2008). Due to its versatility as a thickener, starch is now used more frequently in both industrial and food situations (Sameen et al., 2016). According to Ammar et al. (2009), taro starch (*Colocassia esculenta*) is an excellent source of starch (70%–80%) since it aids in digestion and has favorable qualities for finished products. Because potatoes contain carbohydrates, vitamins A and C, minerals such as iron and potassium, and various fiber ratios; they are also a significant nutritional food (Januário et al., 2017). With all the advantages of starch as a stabilizer in mind, it was intended for the current study to obtain starch from taro (both with and without the application of chemicals) and assess their appropriateness as stabilizers in yogurt production. The study's goals were to optimize the taro concentration for stabilizing the yogurt synthesis and to probe the impact of taro starch on the physiochemical attributes of yogurt.

## 2 MATERIALS AND METHODS

The research was carried out at the Food Safety and Biotechnology Laboratory, as well as other laboratories of Government College University Faisalabad during spring semester of 2021–2022. The study was executed under Complete Randomized Design (CRD) with three replications and two factors. The details of the experiment were described.

### 2.1 Procurement of raw materials

**FIGURE 1:**
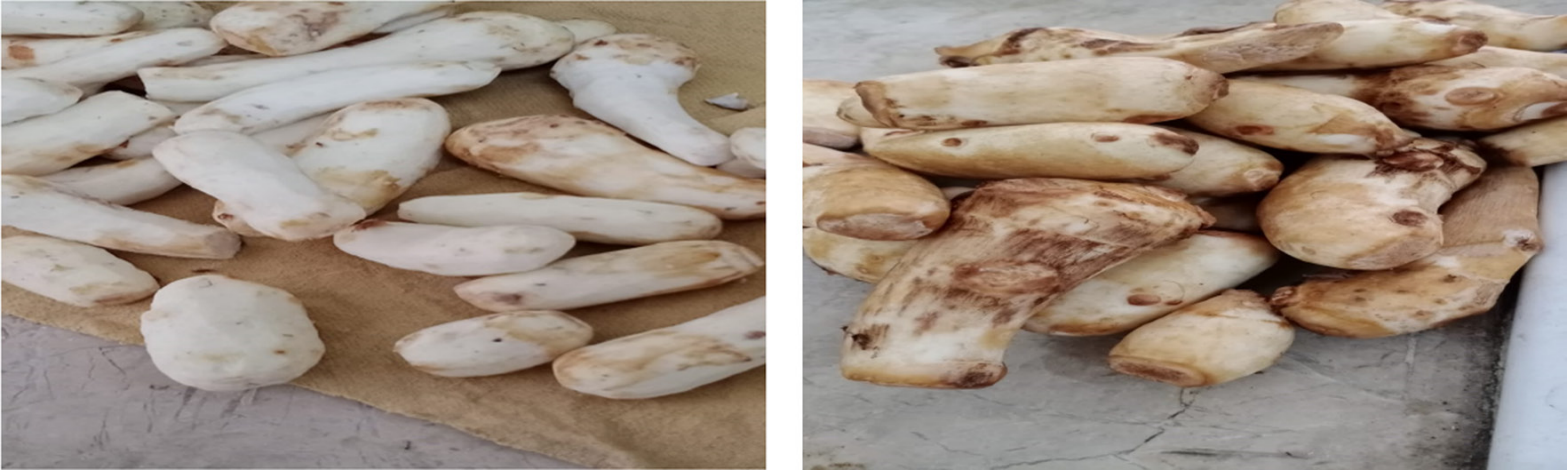
Procurement of taro.

Milk was purchased from a local dairy shop in Faisalabad. Taro and yogurt culture were procured from the local market of Faisalabad city (Figure 1).

### 2.2 Nutritional profile of taro

Taro's nutritional value varies depending on its part. Taro is high in protein, vitamins C and E, riboflavin, phosphorus, thiamine, calcium, and other bioactive compounds (Table 1). The nutritional profile is as follows:

**TABLE 1:** The nutritional profile of taro.

Nutrient (100 g/dry weight)	Crude 1.5 g (taro)
Protein	1.5 g
Ash	1.2 g
Fat	0.2
Carbs	26.46 g
Water	70.64
Energy	112 kcal
Vitamins	
Folates	0.022 mg
Thiamine	0.095 mg
Riboflavin	0.025 mg
Vitamin E	2.36 mg
Vitamin C	4.5 mg
Minerals (mg)	
K	591
Fe	0.550
Mg	33
Na	11
Mn	0.383
Ca	43

### 2.3 Taro starch extraction

**FIGURE 2:**
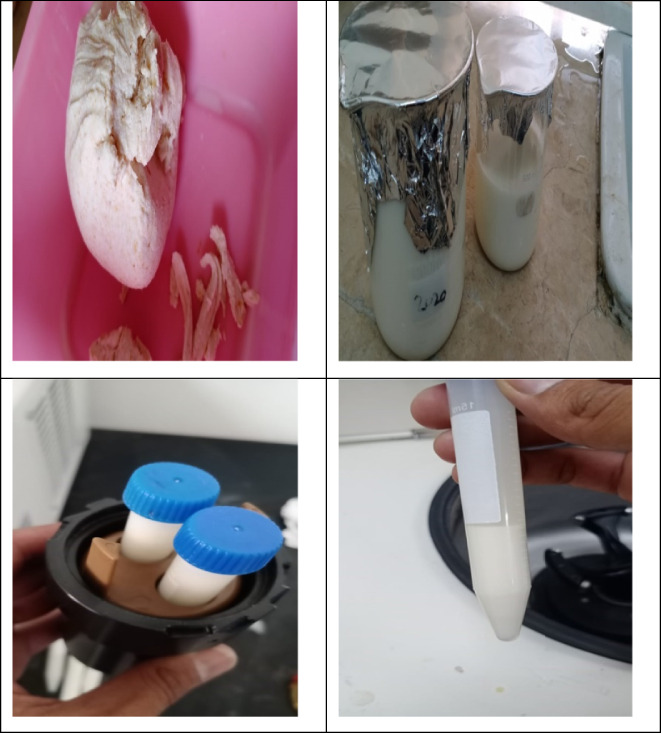
Extraction of taro starch.

**FIGURE 3:**
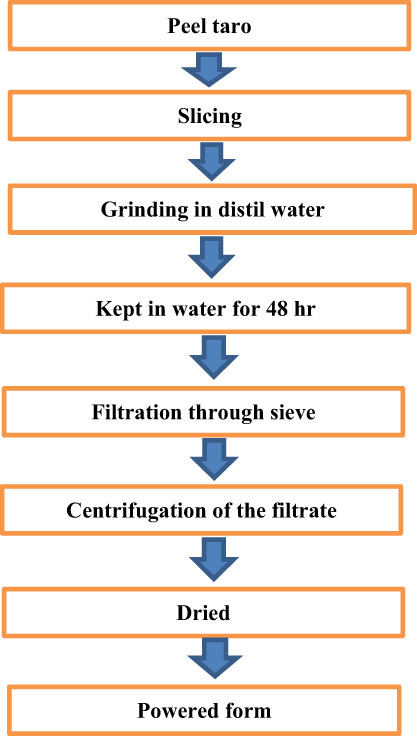
Schematic diagram of taro starch extraction.

The sweet taro pulp was cleaned, peeled, cut, and sieved through a muslin bag. The starch milk was allowed to settle for a while before the supernatant was emptied to produce moist starch cake filtrated. The samples' moist starch cakes were sun‐dried, ground into a fine powder, and stored for further analysis (Oladebeye et al., 2009). After being cleaned, peeled, and clipped, the taro was dehydrated at 50 + 20°C. To get the dry slices through a 500‐m sieve, they were hammer‐milled. For 12 h, 100 g of arvi flour was steeped in a 3‐L water solution. The suspension was filtered through a 150‐m sieve after the slurry had been homogenized with a blender, and it was then given 24 h to settle. The raw starch was collected, cleaned, oven‐dried, and saved for further analysis (Aboubakar et al., 2008; Figures 2 and 3).

### 2.4 Preparation of yogurt

The taro‐fortified yogurt was prepared as per the experimental plan given in Table 1. Yogurt was made by utilizing Malik's technique (2011) from plant sources, chemical‐free starch extraction in this study, taro was added to yogurt at various concentrations shown in Table 1 and stored for 0. 7, and 14 days. There were two factors considered for this study; first one is the different concentrations of taro starch (0%, 0.5%, 1%, 1.5%, 2%, 2.5%, and 3%), and the second one is the storage times (0, 14, and 28 days).

### 2.5 Physiochemical analysis of yogurt

Crude protein, ash, and nitrogen‐free extract were all tested in yogurt (NFE), water, and unrefined fat as defined by AACC (2000) standard.

### 2.6 Moisture

The moisture content of yogurt measured according to method no. 44‐15 A AACC (2000).

### 2.7 Ash determination

Samples were analyzed for ash content by following the AACC (2000) procedure no. 08‐01 as a total inorganic matter. Cookies were subjected to charring process and then ignition was done at 550–600°C for about 5–6 h till graying ash was formed.

### 2.8 Crude fat

The crude fat was calculated using the method (30–25) described in AACC (2000).

### 2.9 Crude protein

Kjeldahl method, as described in AACC (2000) method no. 46‐10, was used to determine the percentage of nitrogen content in each sample.

### 2.10 Nitrogen‐free extracts (NFE)

Particularly, the nitrogen‐free extract was computed using the following equation expressed on the basis of dry matter:

$$\mathrm{NFE}\;\left(\%\right)=100-\left(\text{crude}\;\mathrm{fat}+\mathrm{ash}+\text{crude protein}+\text{crude fiber}\right).$$

### 2.11 Syneresis

The syneresis of starch samples at various storage periods was measured by heating (2% w/v) suspension at 850°C for 30 min and centrifuging at 3200 rpm for 15 min (Singh et al., 2011).

### 2.12 Acidity

The amount of 0.1 N NaOH solution required to neutralize a 10‐g sample of yogurt was used to calculate the acidity values. The total solids were calculated as shown in AOAC (2000).

### 2.13 Viscosity of yogurt

Using the procedure, a Brookfield DV‐E viscometer was used to determine the viscosity of yogurt at 4–6°C. Using the method, different storage periods were determined as free whey (Nafiseh et al., 2008).

### 2.14 Statistical analysis

The collected data were analyzed using a complete randomized design, and their mean comparison was performed using the Tukey honesty test (HSD) at the 1% level of significance (Steel et al., 1997).

## 3 RESULTS AND DISCUSSION

The experiment was carried out in the Department of Food Science's microbiology laboratory at Government College University Faisalabad. The study's goal was to determine the impact of taro starch on yogurt quality (Table 2).

**TABLE 2:** Pasteurized milk analysis.

Ingredient	Unit
Fat	3.58%
pH	4.51
Protein	4.08%
Ash	0.82%
Acidity	0.85

### 3.1 Moisture content percentage of yogurt

The analysis of variance and interaction between different taro starch concentrations and storage days revealed significant differences in yogurt moisture content (Table 3). The results revealed that maximum moisture content individually was attained in 3% added taro starch treatment while the least was assembled in 1.5% addition of taro starch treatment in yogurt. Hence, it was concluded that moisture content start increasing with the increase of storage days as well as increasing the taro starch concentration.

**TABLE 3:** Mean comparison for the nutritional content of yogurt under different concentrations of yogurt at different storage days.

Total starch concentration	Moisture mean ± SD	Crude fat	Crude protein	Ash
T_0_	66.01 ± 1.48^b^	6.11 ± 0.14^d^	4.40 ± 0.24^d^	0.85 ± 0.05^a^
T_1_	66.02 ± 1.48^b^	6.53 ± 0.17^a^	4.97 ± 0.38^a^	0.75 ± 0.02^d^
T_2_	65.94 ± 1.46^b^	6.44 ± 0.16^bc^	4.24 ± 0.23^e^	0.77 ± 0.03^c^
T_3_	64.23 ± 1.45^d^	6.54 ± 0.17^a^	4.37 ± 0.24^d^	0.80 ± 0.04^b^
T_4_	62.43 ± 1.36^e^	6.55 ± 0.17^a^	4.33 ± 0.25^de^	0.75 ± 0.03^cd^
T_5_	65.43 ± 1.46^c^	6.53 ± 0.17^ab^	4.77 ± 0.37^b^	0.82 ± 0.04^b^
T_6_	66.71 ± 1.48^a^	6.36 ± 0.15^c^	4.56 ± 0.26^c^	0.77 ± 0.03^cd^
Mean	65.25 ± 1.46	6.43 ± 0.16	4.52 ± 0.26	0.78 ± 0.03

*Note*: LSD value for Interaction between taro starch concentration× Storage day_(*p* < .01)_ = 0.79.

### 3.2 Fat percentage of yogurt

The analysis of variance and interaction between different concentrations of taro starch and different storage days revealed significant differences in the moisture content of yogurt (Table 3). Fat content can be taken by adding taro starch up to 2% and above this concentration fat percentage started decreasing, on the other hand, fat percentage can be increased by increasing storage days.

### 3.3 Crude protein percentage of yogurt

The interaction of different taro starch concentrations and storage days resulted in significant differences in the crude protein content of yogurt (Table 3). Results showed that maximum crude protein content of 5.07% was attained at 0 days by the application of 1.5% taro starch concentration that was statistically at par with 0.5 and 2.5% taro starch at 14 days, whereas the lowest crude protein of 4.06% was gathered in T_2_S_3_ and T_0_S_1_ treatment by the addition of 1% and no starch application, respectively. It was concluded that crude protein percentage content started decreasing while increasing the storage days.

LSD value for interaction between taro starch and storage days = 0.18.

### 3.4 Ash content of yogurt

Mean sum of square for ash content of yogurt at different concentrations of taro starch and different storage days showed significant variation as well as their interaction effect (Table 3). Percentage of ash content can be taken by adding taro starch up to 2% and above this concentration ash content percentage started decreasing, on the other hand, also ash content percentage started decreasing by increasing storage time.

### 3.5 pH of yogurt

Mean sum of square and main interaction for pH of yogurt under different concentrations of taro starch at different storage times showed significance (Table 4).

**TABLE 4:** Mean comparison for pH, acidity viscosity, and WHC of yogurt under different concentrations of taro starch and storage time.

Taro starch concentration	Storage days		
	S_1_	S_2_	S_3_
pH of yogurt			
t_0_	4.29 ± 0.14^dg^	4.31 ± 0.14^cg^	4.42 ± 0.17^ae^
t_1_	4.42 ± 0.15^ae^	4.13 ± 0.10^g^	4.56 ± 0.19^a^
t_2_	4.51 ± 0.17^ac^	4.34 ± 0.14^bg^	4.39 ± 0.16^af^
t_3_	4.22 ± 0.13^eg^	4.30 ± 0.14^cg^	4.19 ± 0.13^fg^
t_4_	4.54 ± 0.16 ^ab^	4.44 ± 0.15^ad^	4.14 ± 0.12^g^
t_5_	4.25 ± 0.13^dg^	4.19 ± 0.10^fg^	4.21 ± 0.13^fg^
t_6_	4.59 ± 0.17^a^	4.16 ± 0.10^g^	4.24 ± 0.14^dg^
Mean	4.40 ± 0.15	4.26 ± 0.11	4.30 ± 0.15
Acidity of Yogurt			
t_0_	0.78 ± 0.06^gh^	0.78 ± 0.09^gh^	0.89 ± 0.10^cd^
t_1_	0.91 ± 0.80^bc^	0.93 ± 0.12^ab^	0.93 ± 0.11^ab^
t_2_	0.68 ± 0.05^j^ j	0.68 ± 0.08^j^	0.78 ± 0.07^gh^
t_3_	0.76 ± 0.06^hi^	0.77 ± 0.09^gh^	0.85 ± 0.09^e^
t_4_	0.84 ± 0.08^ef^	0.94 ± 0.12^a^	0.91 ± 0.10^bc^
t_5_	0.82 ± 0.07^f^	0.79 ± 0.09^g^	0.74 ± 0.07^i^
t_6_	0.86 ± 0.09 ^de^	0.95 ± 0.13^a^	0.70 ± 0.06^j^
Mean	0.81 ± 0.07	0.83 ± 0.10	0.82 ± 0.08
Viscosity of Yogurt (cp)			
t_0_	714.45 ± 12.43^eg^	689.06 ± 10.53^i^	715.49 ± 10.48^df^
t_1_	689.03 ± 9.43^i^	724.24 ± 17.83^ab^	693.86 ± 9.38^h^
t_2_	726.13 ± 16.43^ab^	715.21 ± 15.83^eg^	724.90 ± 13.48^ab^
t_3_	716.03 ± 13.43^df^	722.83 ± 16.83^bc^	716.41 ± 10.48^df^
t_4_	725.61 ± 15.43^ab^	717.69 ± 14.83^de^	725.94 ± 14.48^ab^
t_5_	718.07 ± 11.43^de^	710.98 ± 13.83^g^	719.72 ± 12.48^cd^
t_6_	712.19 ± 12.34^fg^	693.94 ± 10.63^h^	727.30 ± 15.47^a^
Mean	714.50 ± 12.43	710.56 ± 13.83	717.66 ± 11.48
Water‐holding capacity of yogurt			
t_0_	67.56 ± 5.15^e^	63.75 ± 4.10^f^	66.89 ± 5.10^e^
t_1_	63.77 ± 5.11^f^	74.73 ± 6.72^b^	73.83 ± 6.13^b^
t_2_	73.79 ± 7.10^bc^	67.83 ± 4.14^e^	67.75 ± 5.43^e^
t_3_	81.53 ± 8.14^a^	81.49 ± 7.62^a^	63.86 ± 5.10^f^
t_4_	66.87 ± 5.18^e^	72.43 ± 6.10^c^	73.83 ± 6.13^b^
t_5_	74.76 ± 7.14^b^	69.86 ± 5.81^d^	81.65 ± 8.12^a^
t_6_	67.51 ± 5.12^e^	66.44 ± 4.51^e^	66.97 ± 5.1^e^
Mean	70.83 ± 6.14	70.93 ± 5.92	70.68 ± 6.10

*Note*: LSD value for Taro starch = 0.1219; LSD value for Storage = 0.0798.

### 3.6 Acidity of yogurt

Mean sum of square and main interaction for acidity of yogurt under different concentrations of taro starch at different storage times showed significance (Table 4). Khalifa et al. (2011) also revealed that the acidity of yogurt gradually increased with storage. Andic et al. (2013) and Anwer et al. (2013) found a link between lactic acid production from lactose by lactic acid bacteria and the increase in acidity of yogurt during storage.

### 3.7 Viscosity of yogurt (cp)

Mean sum of square and main interaction for viscosity of yogurt under different concentrations of taro starch at different storage times showed statistical significance (Table 4). The results in Table 4 are found to be consistent with Eissa et al. (2011), who reported that viscosity decreases with storage due to increased acidity and syneresis.

### 3.8 Water‐holding capacity of yogurt

The mean sum of the square and main interaction for water‐holding capacity of yogurt under different concentrations of taro starch at different storage times showed statistical significance (Table 4). According to Sakandar et al. (2014), water‐holding capacity decreases with storage due to increased acidity and syneresis. Table 4 demonstrates greater viscosity and water‐holding capacity, as well as less syneresis, when compared to the gelatin‐containing sample.

### 3.9 Syneresis of yogurt

**FIGURE 4:**
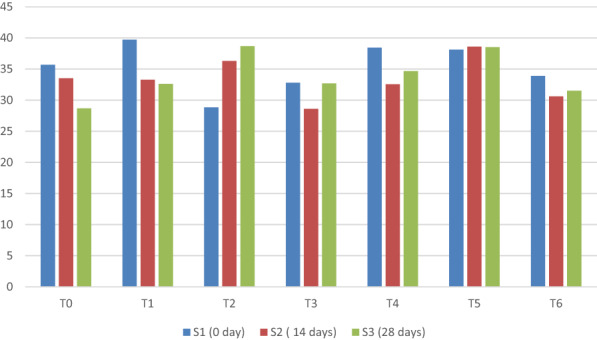
Mean comparison for syneresis of yogurt under different concentrations of taro starch at different storage days.

Mean sum of square and main interaction for syneresis of yogurt under different concentrations of taro starch at different storage times showed statistical significance (Figure 4). The results shown in Figure 4 are consistent with the findings of Chye et al. (2012), who discovered that the synergy of yogurt increases with storage time.

### 3.10 Yogurt texture profile (TPC) (CFU/mL)

**FIGURE 5:**
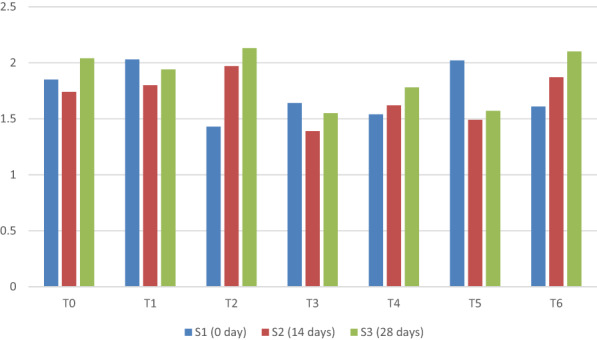
Mean comparison for total plate count of yogurt under different concentrations of taro starch concentration at different storage days.

The mean sum of a square and the main interaction for the total plate count of yogurt under different concentrations of taro starch at different storage times showed statistical significance (Figure 5).

### 3.11 Aroma of yogurt

**FIGURE 6:**
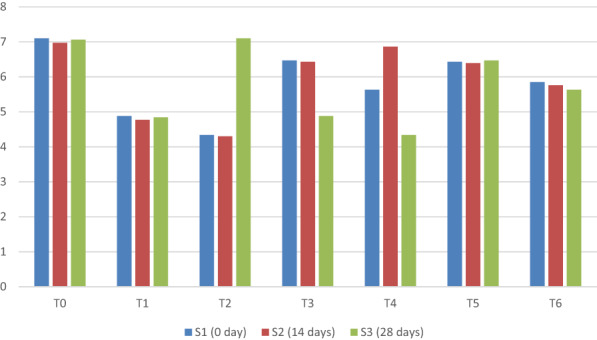
Mean comparison for aroma of yogurt under different concentrations of taro starch and varying storage days.

A large number of volatile bacterial metabolites contribute to the aroma of solid products, some of which are lactic acid fermentation by‐products or produced by other reaction mechanisms. The aroma and flavor of yogurt are mainly due to nonvolatile, volatile acids, and carbonyl compounds. The results given in Figure 6 describes the statistical profile for aroma of probiotic yogurt prepared by milk supplemented with different concentrations of taro starch. The results for variance analysis showed significant variance for yogurt aroma (*p* < .01). The statistical analysis revealed significant differences between all samples. Salwa et al. (2004) also found that the chemical and sensory properties of yogurt deteriorated over time.

### 3.12 Taste of yogurt

**FIGURE 7:**
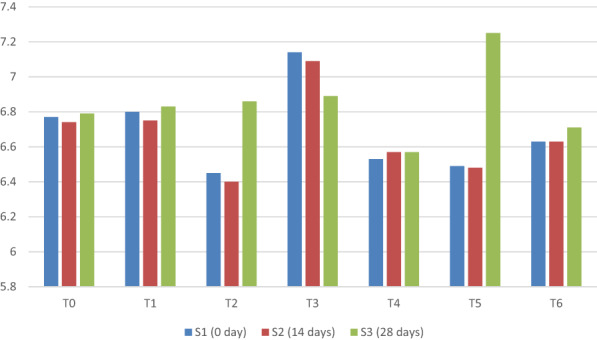
Mean comparison for taste of yogurt under different concentrations of taro starch and different storage periods.

The sensation of flavor perceived in the mouth and throat upon contact with a substance is referred to as taste. It is a main sensory property of the product. It describes the better acceptance of products by people who are more conscious about the taste of food. Greek yogurt or a nondairy, plain yogurt (e.g., coconut, almond, or soy) alternatives taste pretty sour and tart. That sour flavor can be off‐flavor, so many brands have flavored alternatives to cover that sour flavor. Figure 7 shows the results of the analysis of variance related to the taste of yogurt prepared with milk and different concentrations of taro starch. Figure 7 indicates the significant statistical results of the yogurt with different concentrations of taro starch and different storage periods.

### 3.13 Flavors of yogurt

**FIGURE 8:**
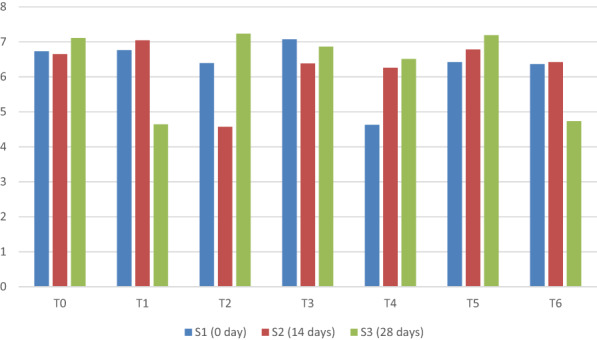
Mean comparison for flavors of yogurt under different concentrations of taro starch concentrations and different storage periods.

Yogurt cultures are known for their bacteria. In milk, sugar fermentation produces the milk acid that works on milk protein, giving yogurt the texture and tart flavor. Yogurt is defined by fermentation with mixed crops as a product resulting from milk, the texture, the surface, and taste and health effects linked to yogurt. Figure 8 describes the results of flavor profile of probiotic yogurt prepared from milk under different concentrations of taro starch and different storage days. The analysis of variance results revealed significant differences (*p* < .01) in flavor content in yogurt.

### 3.14 Overall adaptability

**FIGURE 9:**
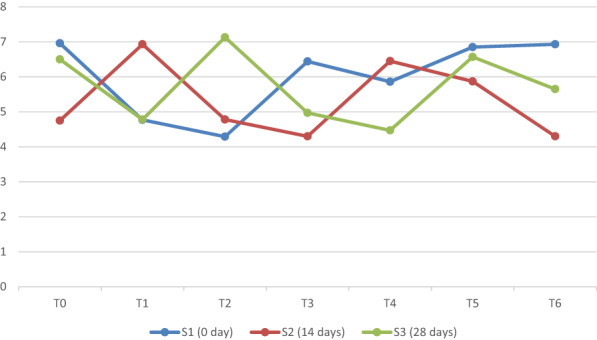
Mean comparison for overall adaptability of yogurt under different concentrations of taro starch under varying storage days.

Acceptableness is the characteristic of something which is for some purpose subject to acceptance. Anything is acceptable if it is enough to fulfill the purpose it is for, even if it is far less useful than the ideal example. Food acceptability relates directly to the food interaction at a particular time. Customer characteristics, sensory characteristics, e.g., taste, texture, flavor, and aroma of food, and the ‘feeling good’ factor include factors that affect food acceptability as covered by the article. Figure 9 describes the results for overall acceptability of yogurt under different concentrations of taro starch and different storage days. The acceptability of probiotic yogurt differed significantly (*p* < .01).

## 4 DISCUSSION

The human body needs some essential nutrients like water, protein, fat, and carbohydrates and milk contains the entire essentials as well as the minor nutrients of minerals, enzymes, vitamins, and dissolved gases. Milk is easily perishable because the nutrients present in milk act as a hospitable environment for the growth of microorganisms. One strategy to deal with this is to diversify milk processing by using fermentation technology to produce yogurt.

Yogurt is a dairy product with a milk casein coagulation pH of approximately 4.6. *Lactobacillus bulgaricus* and *Streptococcus thermophilus* are the two types of lactic acid bacteria that are typically employed in the production of yogurt. Many probiotic BAL species, such as L. acidophilus, can survive in low gastric pH and adhere to or colonize the intestine, improving function and nutritional value. The current study was executed to enhance the shelf life, aroma, texture, flavor, color, and viscosity. Different types of starches are used for enhancing the functional characteristics of yogurt synthetically and naturally. The experiment was designed with a completely randomized design and factor distribution across three replications. There were two factors considered for this study; first one is the different concentrations of taro starch (0%, 0.5%, 1%, 1.5%, 2%, 2.5%, and 3%), and the second one is the storage times (0, 14, and 28 days). HSD test was employed for mean comparison and the level of significance was kept at 1%. The results of the study are given below.

Data regarding the moisture content of yogurt showed that maximum content was attained by that treatment where 0.5% taro starch was added and stored for 0 days while the lowest moisture content was present in T_3_S_2_ where 1.5% taro starch was added and stored for 14 days. The maximum fat of 6.73% was recorded in the 1.5 taro starch added sample and remained for 0 days for storage, whereas the lowest fat percentage was noted in the T_o_S_1_ sample where no taro starch was added and stored for 0 days. The maximum acidity of yogurt was noted when 3% taro starch was added and stored for 14 days. The acidity of yogurt was increased with the increase of taro starch and increasing the storage days. Maximum protein content was achieved in T_1_S_1_ treatment where only 0.5% taro starch was added and stored for 0 days after some extent of taro starch, increasing the taro starch concentration which ultimately loses the protein content of yogurt. The maximum syneresis was recorded in T_1_S_1_ where 0.5% taro starch was added and stored for 0 days, while the minimum syneresis was taken in T_3_S_2_ where 1.5% taro was added as a supplement and stored for 14 days. The maximum viscosity was recorded in the T4 treatment with 2% taro starch and the lowest viscosity was recorded in the T_4_ treatment with 0.5% taro starch. The concentration of taro starch increased the viscosity of yogurt. Water‐holding capacity was maximum in the T_3_ treatment where 1.5% taro starch was added and the least water‐holding capacity was recorded in that treatment where no taro starch was added. It was concluded that water‐holding capacity can be enhanced by the addition of some taro starch and holding capacity can be decreased while lowering the taro concentration.

## 5 CONCLUSION

Yogurt is an ancient milk product around the world and is widely used in the world due to its numerous characteristics and high nutritional attributes. The main issue faced by the yogurt industry during production and maintenance is ensuring yogurts’ stabilization and consistency. Yogurt stabilizers are used to upgrade the flavor, texture, and appearance of yogurt, while reducing syneresis. The overall results of the study showed that by adding 2% taro starch concentration, aroma, taste, water‐holding capacity, viscosity, fat, and protein content can be enhanced, and by increasing storage days of yogurt, sensory properties start decreasing, as well as water‐holding capacity, and yogurt can be stored for up to 14 days by adding 2% taro starch.

## AUTHOR CONTRIBUTIONS

**Muhammad Shaheryar:** Writing – original draft (equal). **Muhammad Afzaal:** Supervision (equal). **Farhana Nosheen:** Supervision (equal). **Ali Imran:** Methodology (equal); visualization (equal). **Fakhar Islam:** Methodology (equal); writing – review and editing (equal). **Rabia Noreen:** Validation (equal). **Umber Shehzadi:** Formal analysis (equal). **Mohd Asif Shah:** Visualization (equal). **Adil Rasool:** Software (equal).

## FUNDING INFORMATION

The authors declare that no funds, grants, or other support were received during the preparation of this manuscript.

## CONFLICT OF INTEREST STATEMENT

The authors that they have declare no conflict of interest.

## ETHICS STATEMENT

The study does not involve any human or animal testing.

## CONSENT TO PARTICIPATE

All the co‐authors are willing to participate in this manuscript.

## CONSENT FOR PUBLICATION

All authors are willing for publication of this manuscript.

## Data Availability

The data that support the findings of this study are available from the corresponding author upon request.

## References

[fsn33358-bib-0001] AACC (Ed.). (2000). Approved Methods of the American Association of Cereal Chemists (10th ed.). AACC.

[fsn33358-bib-0002] Aboubakar, Y. N. , Njintang, J. S. , & Mbofung, C. M. F. (2008). Physicochemical, thermal properties and microstructure of six varieties of taro (*Colocasia sculenta* L. Schott) flours and starches. Journal of Food Engineering, 86, 294–305.

[fsn33358-bib-0003] Alakali, J. , Okonkwo, T. , & Iordye, E. E. (2008). Effect of stabilizers on the physico‐chemical and sensory attributes of thermized yogurt. African Journal of Biotechnology, 7, 7–15.

[fsn33358-bib-0004] Ammar, M. S. , Hegazy, A. E. , & Bedeir, S. H. (2009). Using of taro flour as partial substitute of wheat flour in bread making. World Journal of Dairy & Food Sciences, 4, 94–99.

[fsn33358-bib-0005] Andic, S. , Boran, G. , & Tuncturk, Y. (2013). Effects of carboxyl methyl cellulose and edible cow gelatin on physico‐chemical, textural and sensory properties of yoghurt. International Journal of Agriculture and Biology, 15, 245–251.

[fsn33358-bib-0006] Anwer, M. , Ahmad, S. , Sameen, A. , & Ahmed, S. (2013). Effect of different heating temperatures on the rheological properties of lactic gel made from buffalo milk. Journal of Food Chemistry and Nutrition, 1, 33–41.

[fsn33358-bib-0007] Arioui, F. A. , Saada, D. , & Cheriguene, A. (2017). Physicochemical and sensory quality of yogurt incorporated with pectin from peel of Citrus sinensis. Food Science & Nutrition, 5, 358–364. 28265371 10.1002/fsn3.400 PMC5332253

[fsn33358-bib-0008] Chye, S. J. , Ahmad, R. , & Aziah, A. A. N. (2012). Studies on the physicochemical and sensory characteristics of goat's milk incorporated with tropical fruit purees. International Food Research Journal, 19(4), 1387–1392.

[fsn33358-bib-0009] Eissa, E. A. , Babikerand, E. E. , & Yagoub, A. A. (2011). Physicochemical, microbiological and sensory properties of Sudanese yoghurt (zabadi) made from goat's milk. Animal Production Science, 51, 53–59.

[fsn33358-bib-0010] El‐Said, M. M. , Haggag, H. F. , El‐Din, H. M. F. , & Gad, A. S. (2014). Antioxidant activities and physical properties of stirred yogurt fortified with pomegranate peel extracts. Annals of Agricultural Sciences, 59, 207–212.

[fsn33358-bib-0011] Gemechu, T. (2015). Review on lactic acid bacteria function in milk fermentation and preservation. African Journal of Food Science, 9, 70–175.

[fsn33358-bib-0012] Hematyar, N. , Samarin, A. M. , Poorazarang, H. , & Elhamirad, A. H. (2012). Effect of gums on yogurt characteristics. World Applied Sciences Journal, 20, 661–665.

[fsn33358-bib-0013] Imeson, A. (1999). Thickening and gelling agents for foods (2nd ed.). Aspen Publishers, Inc.

[fsn33358-bib-0014] Islam, F. , Amer Ali, Y. , Imran, A. , Afzaal, M. , Zahra, S. M. , Fatima, M. , Saeed, F. , Usman, I. , Shehzadi, U. , Mehta, S. , & Shah, M. A. (2023). Vegetable proteins as encapsulating agents: Recent updates and future perspectives. Food Science & Nutrition, 11, 1705–1717. 10.1002/fsn3.3234 37051354 PMC10084973

[fsn33358-bib-0015] Januário, J. G. B. , da Silva, I. C. F. , De Oliveira, A. S. , De Oliveira, J. F. , Dionísio, J. N. , Klososki, S. J. , & Pimentel, T. C. (2017). Probiotic yogurt flavored with organic beet with carrot, cassava, sweet potato or corn juice: Physicochemical and texture evaluation, probiotic viability and acceptance. International Food Research Journal, 24, 359–366.

[fsn33358-bib-0016] Khalifa, E. A. , Elgasim, A. E. , Zaghloul, A. H. , & Mahfouz, M. B. (2011). Application of inulin and mucilage as stabilizers in yoghurt production. American Journal of Food Technology, 6, 31–39.

[fsn33358-bib-0017] Lucey, J. (2002). Formation and physical properties of Milk protein gels. Journal of Dairy Science, 85, 281–294. 11913691 10.3168/jds.s0022-0302(02)74078-2

[fsn33358-bib-0018] Malik, A. H. , Anjum, F. M. , Sameen, A. , Khan, M. I. , & Sohaib, M. (2012). Extraction of starch from water chestnut (*Trapa bispinosa* Roxb) and its application in yogurt as a stabilizer. Pakistan Journal of Food Sciences, 22, 209–218.

[fsn33358-bib-0019] Maryam, T. M. , Nafiseh, F. , Caro, L. , & Fattaneh, T. (2009). Artificial neural network weights optimization based on imperialist competitive algorithm . In 7th international conference on computer science and information technologies (CSIT 2009).

[fsn33358-bib-0020] McKinley, M. C. (2005). The nutrition and health benefits of yogurt. International Journal of Dairy Technology, 58, 1–12.

[fsn33358-bib-0021] Mistry, V. , & Hassan, H. (1992). Manufacture of nonfat yogurt from a high Milk protein powder. Journal of Dairy Science, 75, 947–957. 1578031 10.3168/jds.S0022-0302(92)77835-7

[fsn33358-bib-0022] Nikoofar, E. , Hojjatoleslami, M. , & Shariaty, M. A. (2013). Surveying the effect of quince seed mucilage as a fat replacer on texture and physicochemical properties of semi fat set yogurt. International Journal of Farming and Allied Sciences, 2, 861–865.

[fsn33358-bib-0023] Oladebeye, A. O. , Oshodi, A. A. , & Oladebeye, A. A. (2009). Physicochemical properties of starches of sweet potato (Ipomeabatata) and red cocoyam (*Colocasia esculenta*) cormels. Pakistan Journal of Nutrition, 8, 313–315.

[fsn33358-bib-0024] Pang, Z. , Deeth, H. , Yang, H. , Prakash, S. , & Bansal, N. (2017). Evaluation of tilapia skin gelatin as a mammalian gelatin replacer in acid milk gels and low‐fat stirred yogurt. Journal of Dairy Science, 100, 3436–3447. 28284688 10.3168/jds.2016-11881

[fsn33358-bib-0025] Pundir, R. K. , Rana, S. , Kashyap, N. , & Kaur, A. (2013). Probiotic potential of lactic acid bacteria isolated from food samples: An in vitro study. Journal of Applied Pharmaceutical Science, 3, 85–93.

[fsn33358-bib-0026] Sakandar, H. A. , Imran, M. , Huma, N. , Ahmad, S. , Aslam, H. K. W. , Azam, M. , & Shoaib, M. (2014). Effects of polymerized whey proteins isolates on the quality of stirred yoghurt made from camel milk. Journal of Food Processing & Technology, 5(7), 1.

[fsn33358-bib-0027] Salwa, A. A. , Galal, E. A. , & Neimat, A. E. (2004). Carrot yoghurt: Sensory, chemical, microbiological properties and consumer acceptance. Pakistan Journal of Nutrition, 3(6), 322–330.

[fsn33358-bib-0028] Sameen, A. , Sattar, M. U. , Javid, A. , Ayub, A. , & Khan, M. I. (2016). Quality evaluation of yogurt stabilized with sweet potato (*Ipomoea batatas*) and taro (*Colocassia esculenta*) starch. International Journal of Food and Allied Sciences, 2, 23–29.

[fsn33358-bib-0029] Singh, S. , Singh, N. , Ezekiel, R. , & Kaur, A. (2011). Effects of gamma irradiation on morphological, structural, thermal and rheological properties of potato starch. Carbohydrate Polymers, 83, 1521–1528.

[fsn33358-bib-0030] Steel, R. G. D. , Torrie, J. H. , & Dicky, D. A. (1997). Principles and procedures of statistics. A biometrical approach (3rd ed.). McGraw Hill Book Co. Inc.

[fsn33358-bib-0031] Sulistyowati, P. V. , Kendarini, N. , & Respatijarti, E. (2014). Observasi Keberadaan Tanaman Talas‐Talasan Genus Colocasia Dan Xanthosoma Di Kec. Kedungkandang Kota Malang Dan Kec. *Ampelgading Kab. Malang* . Jurnal Produksi Tanaman, 2, 86–93.

[fsn33358-bib-0032] Zhao, Q. , Zhao, M. , Yang, B. , & Cui, C. (2009). Effect of xanthan gum on the physical properties and textural characteristics of whipped cream. Food Chemistry, 116, 624–628.

[fsn33358-bib-0033] Zubair, M. W. , Imran, A. , Islam, F. , Afzaal, M. , Saeed, F. , Zahra, S. M. , Akhtar, M. N. , Noman, M. , Ateeq, H. , Aslam, M. A. , Mehta, S. , Shah, M. A. , & Awuchi, C. G. (2023). Functional profile and e. Food Science & Nutrition, 1–10. 10.1002/fsn3.3357 PMC10261768 37324900

